# Contributions of natural and anthropogenic radiative forcing to mass loss of Northern Hemisphere mountain glaciers and quantifying their uncertainties

**DOI:** 10.1038/srep29723

**Published:** 2016-07-20

**Authors:** Yukiko Hirabayashi, Kazunari Nakano, Yong Zhang, Satoshi Watanabe, Masahiro Tanoue, Shinjiro Kanae

**Affiliations:** 1 Institute of Engineering Innovation, The University of Tokyo, 2-11-16 Yayoi, Bunkyo-ku, Tokyo 113-8656, Japan; 2 Department of Civil Engineering, Tokyo Institute of Technology, 2-12-1, Ookayama, Meguro-ku, Tokyo 152-8552, Japan

## Abstract

Observational evidence indicates that a number of glaciers have lost mass in the past. Given that glaciers are highly impacted by the surrounding climate, human-influenced global warming may be partly responsible for mass loss. However, previous research studies have been limited to analyzing the past several decades, and it remains unclear whether past glacier mass losses are within the range of natural internal climate variability. Here, we apply an optimal fingerprinting technique to observed and reconstructed mass losses as well as multi-model general circulation model (GCM) simulations of mountain glacier mass to detect and attribute past glacier mass changes. An 8,800-year control simulation of glaciers enabled us to evaluate detectability. The results indicate that human-induced increases in greenhouse gases have contributed to the decreased area-weighted average masses of 85 analyzed glaciers. The effect was larger than the mass increase caused by natural forcing, although the contributions of natural and anthropogenic forcing to decreases in mass varied at the local scale. We also showed that the detection of anthropogenic or natural influences could not be fully attributed when natural internal climate variability was taken into account.

Decreases in glacier mass have been observed in many regions over the last several decades^1^,^2^. The negative mass balances of glaciers reflect characteristics of the surrounding climate, such as surface temperature and precipitation. In many regions, the observed upward trend in surface temperature cannot be explained only by natural internal climate variability^3^. On the other hand, increases in greenhouse gases are expected to cause enhanced horizontal transport of water vapor, leading to an increase in precipitation at high latitudes where many glaciers are located^4^. Since the combined changes in temperature and precipitation would affect glaciers, identifying a probable cause of the recent losses in glacier mass is an important area of research.

Most climatic attribution studies have focused on single-step variables, such as surface temperature^3^,^5^, that are modeled explicitly in a General Circulation Model (GCM) as directly responding to external forcing to the climate system (e.g., volcanic activity, solar activity, and artificial CO_2_ emissions). However, in recent years, attribution assessment has also been applied to multi-step variables that are closely related to changes in single-step climate variables (e.g., evapotranspiration^6^, Arctic sea ice^7^ and Northern Hemisphere spring snow cover^8^). Because changes in glacier mass are often highly affected by surface temperature and precipitation (except for glaciers largely affected by surging or calving processes), the recent negative mass changes of glaciers could possibly be attributed to historical increases in anthropogenic CO_2_ emissions. However, it has not yet been possible to detect such an influence due to the difficulty of obtaining long-term direct observations of glacier mass.

One recent study^9^ indicated that some mass loss can be attributed to anthropogenic causes; the authors used both observed and modeled glacier mass balances forced by climate variables obtained from historical GCM experiments under different external forcings. However, due to the limited data available, it remains unclear whether past trends of glacier mass loss are within the range of natural internal climate variability. Reliably estimating the amplitude of natural climate variability is critical to understanding anthropogenic effects on climate, since this variability can obfuscate climate trend attribution. Although the aforementioned previous study compared glacier masses from simulations forced by all known external forcing and only natural forcing, it would be useful to analyze an additional simulation with only anthropogenic forcing to quantify its contributions to past glacier mass.

In this study, we tried to detect and attribute changes in the mass of mountain glaciers (excluding tidewater glaciers, ice caps, and ice sheets) to determine whether recent prominent mass losses have been caused by anthropogenic climate change. To establish the most likely sources of the detected changes with some level of confidence, we applied an optimal fingerprinting method^10^,^11^, which has been frequently used to detect and attribute effects of climate change. We conducted two types of analyses. The first was an ordinary detection and attribution analysis of a fingerprinting method using direct glacier mass measurements obtained between 1949 and 2003 from the Glacier Mass Balance Bulletin^12^ as well as a set of modeled glacier mass using a global glacier model, HYOGA2^13^, forced by the output of GCM experiments. This analysis was conducted for five glaciers in Western Europe, where direct long-term observations of mass balance were available (Supplementary Table S1). Due to the limited availability of direct long-term glacier measurements, an additional analysis of 85 widely distributed glaciers was performed with a modeled glacier mass forced by the same set of GCM experiments and reconstructed glacier masses, using HYOGA2 forced by observation-based climate data.

The climate experiments were conducted using HYOGA2 forced by daily precipitation and temperature outputs of the latest GCMs implemented in phase 5 of the Coupled Model Intercomparison Project^14^ (CMIP5, Supplementary Table S2). Biases in the GCMs were corrected against the historical climate data (see Methods “Model data processing and HYOGA2 simulation” for details). We used GCM experiments for 1949–2003 forced with all known forcing including combined historical natural (solar and volcanic) plus anthropogenic greenhouse gas forcings (ALL; 14 GCMs), historical anthropogenic greenhouse gas forcing only (GHG; 7 GCMs), and natural forcing only (NAT; 7 GCMs) to calculate glacier mass changes with and without anthropogenic forcing. The additional GHG analysis enables us to attribute the effects of anthropogenic radiation forcing on past glacier mass changes. Because observed records of glacier mass are usually too short to estimate the chaotic nature of climate systems, we used unforced control GCM simulations (CTL; 160 non-overlapping 55-year segments from 14 GCMs) (Supplementary Table S3) to obtain the uncertainty range of the results. We assumed that the control run of a climate model could estimate the distribution of natural internal climate variability^10^.

## Results

**Figure 1:**
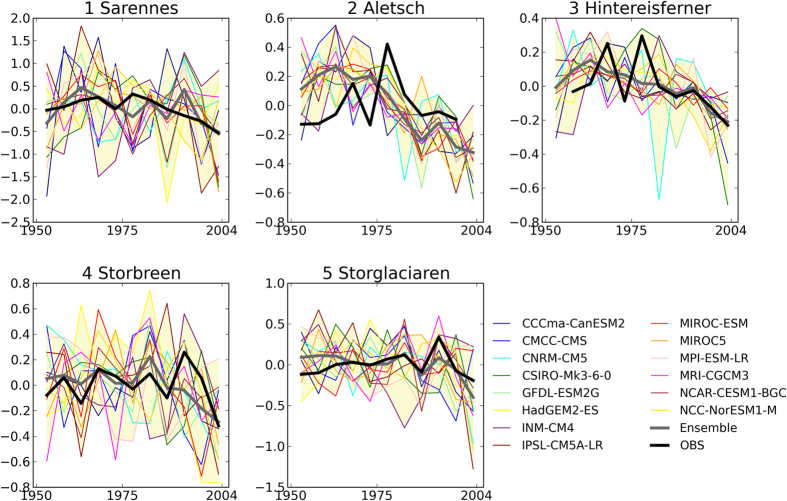
Time series of 5-year mean annual mass balance anomalies of five monitored glaciers from the 1950 to 1980 mean (m). Model simulations with all known forcings (ALL; colored line), their ensemble means (thick gray line), and observations (thick black line). The shading denotes the maximum and minimum range of all modeled values. This figure was created using Python 2.7.2.

We analyzed five monitored glaciers in Western Europe. Figure 1 shows the simulated and observed changes in annual glacier mass from 1949 to 2003. Similar to previous studies^15^, we eliminated discrepancies between inter-annual glacier mass variations from GCM experiments and observations by applying a 5-year average to anomalies from the 1950 to 1980 mean. Although the GCMs had variable results, all of the five glaciers exhibited decreasing trend for both observations and the multi-model ensemble of ALL simulations, particularly after the 1980s.

**Figure 2:**
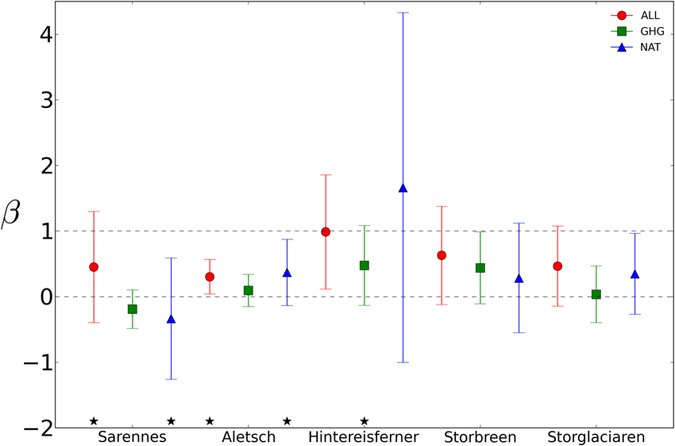
Results of optimal detection analyses of glacier mass balance over the five monitored glaciers. Best estimates (data points) and 95% confidence intervals of beta (error bars) are displayed for fingerprint analyses for ALL (red), GHG (green), and NAT (blue) (see Methods for details). The two gray dashed horizontal lines represent zero and unity. An asterisk indicates failure of the residual consistency test. This figure was created using Python 2.7.2.

Then, a multivariate regression method of the optimal fingerprinting was applied to the observed and modeled glacier masses to statistically assess the detection quality, taking into account the natural internal climate variations derived from CTL simulations. Of the 15 experiments (ALL, GHG, and NAT at 5 glaciers), 10 passed the residual consistency test^10^ (Fig. 2) (see Methods “Detection and attribution” and “Residual consistency test” for details), indicating that the control simulation was long enough to produce internal climate variability. The best estimates of the regression coefficients obtained from the ALL simulations were >0 at all five analyzed glaciers (Fig. 2). This indicates that HYOGA2, forced by historical GCM experiments with ALL captured past changes similar to the observations. The regression coefficients of ALL simulations were <1 at all of the glaciers except for the Hinterisferner glacier, indicating that ALL simulations overestimated the changes of these glaciers. Error bars in Fig. 2 show 95% confidence intervals (CIs) for the optimal ordinary least squares (OLS)^6^,^10^ regression coefficients using CTL experiments (see Methods “Detection and attribution” for details). At the Aletch and Hintereisferner glaciers, the hypothesis of a zero scaling factor is rejected, indicating that the ALL simulation was consistent with observations.

To attribute potential sources of change to externally forced climate change signals, we conducted the same analysis for the GHG and NAT simulations. The results showed that the contribution of anthropogenic or natural forcing varied among glaciers (Supplementary Figs S4 and S5; Fig. 2). For the Aletch, Hintereisferner, and Storbreen glaciers, the GHG regression coefficients were between 0 and 1, indicating that GHG forcing contributed to the observed mass losses and that anthropogenic radiation forcing has a negative effect on mass at these glaciers. On the other hand, GHG regression coefficients at Sarennes and Storglaciaren were negative or close to 0, respectively. The regression coefficient at Sarennees was also negative in NAT. This indicates that external forcing (GHG or NAT) contributes to a trend opposite that of observations. In the other three glaciers, NAT simulations had values between 0 and 1, indicating that natural radiative forcing contributed to the observed negative change.

**Figure 3:**
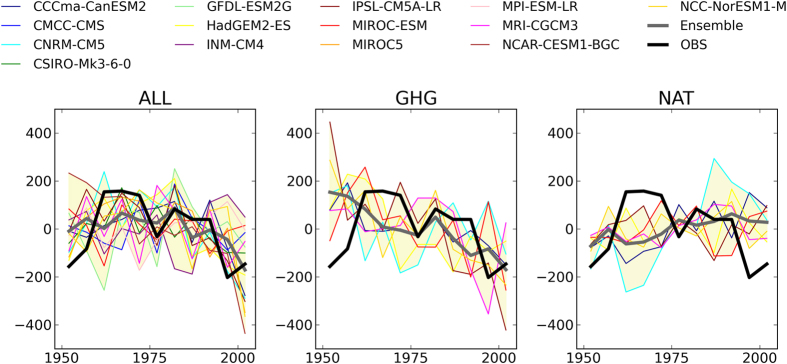
Same as Fig. 1 but for area-averaged mass balance anomalies of 85 reconstructed glaciers for the ALL (left), GHG (middle), and NAT (right) simulations. This figure was created using Python 2.7.2.

Because analyses of direct mass observations were limited to Western Europe, we conducted additional analyses on 85 glaciers widely distributed in the Northern Hemisphere (Fig. S1; Table S4). The area-weighted average of global mass changes in observations and the ALL experiment showed a negative trend, in particular after the 1980s (Fig. 3). The ensemble mean of global mass changes in GHG also showed a similar negative trend. On the other hand, the ensemble mean in NAT showed a positive trend, indicating that the observed negative trend may not be explained without considering anthropogenic forcing.

**Figure 4:**
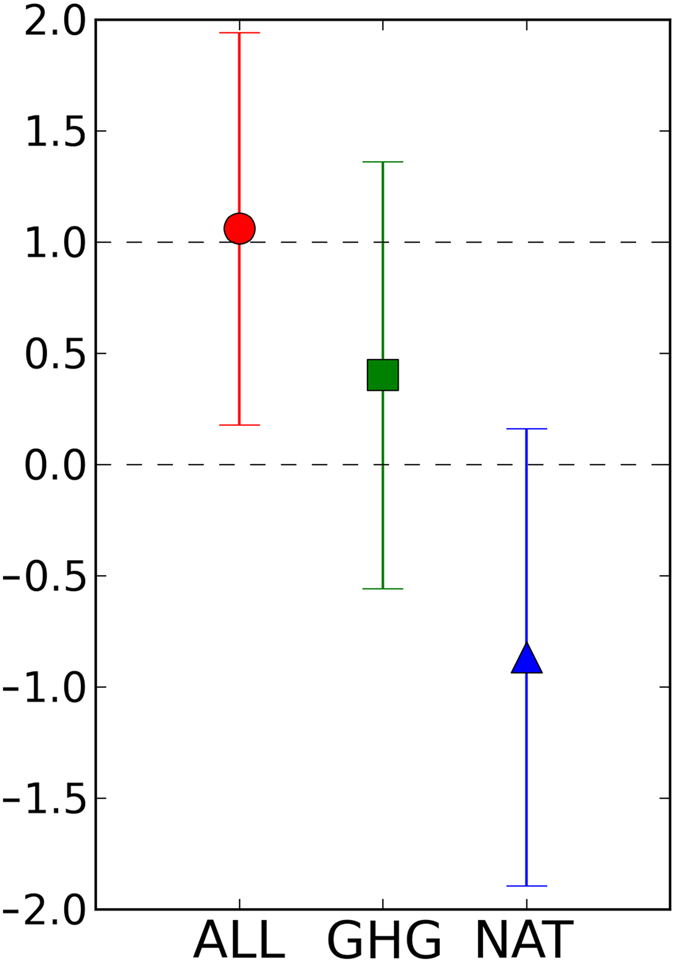
Same as Fig. 2 but for area-averaged mass balance of 85 reconstructed glaciers. This figure was created using Python 2.7.2.

The difference in the contribution of external forcings to mass change is also obvious in the best estimates of the regression coefficients of the optimal fingerprinting method for the three experiments (ALL, GHG, and NAT) of the area-weighted mass changes of the 85 glaciers (Fig. 4). The regression coefficient for the ALL experiment (1.06) indicates that the model captured the observed mass change, fairly well, including the negative change after the 1980s. The regression coefficient obtained when using GHG fingerprints was positive, suggesting that anthropogenic radiative forcing influenced the decrease in glacier masses. On the other hand, the regression coefficient from the NAT fingerprint was negative, indicating that natural forcing counteracts the observed mass changes.

The 95% CI of the regression coefficients returned a value higher than zero in the ALL experiments (error bars in Fig. 4). Therefore, we rejected the hypothesis that external forcing (both anthropogenic and natural radiation forcing) have no effects on the observed glacier mass. On the other hand, the GHG and NAT error bars included zero, so we did not reject the hypothesis of a zero scaling factor (at the 5% level). Thus, the effects of anthropogenic or natural influences remain unclear.

## Discussion

The results indicated that although all of the glaciers in ALL simulations had changes similar to the observations (scaling factor >0.0), the contributions of anthropogenic and natural external forcings varied among glaciers (Fig. 2). This highlights the difficulty of evaluating the contributions of these factors using the fingerprinting method at the local level. Previous studies have indicated that GCMs may fail to replicate observed changes when small areas are considered^15^,^16^. Despite limitations at local scales, we confirmed that these methods maintained the signal (positive or negative) of the external forcing contribution when a reconstructed glacier mass was used instead of an observed mass (Supplementary Fig. S3).

Similar to a previous study^9^, the calculated glacier mass increased in the NAT simulations (Fig. 3), possibly due to the effects of low insolation (e.g., volcanic eruptions or changes in solar activity). The area-weight mean mass of 85 glaciers in the GHG simulations decreased (Fig. 3). The fingerprinting results showed the contribution of anthropogenic greenhouse emissions to glacier mass loss (Fig. 4). Recent increases in surface temperature due to anthropogenic forcing may increase glacier melting. The additional GHG experiment supported the effects of anthropogenic greenhouse gases on glacier mass loss suggested by a previous study, which compared modeled mass changes in ALL and NAT experiments^9^.

The optimal fingerprinting method provided an attribution uncertainty range using the long-term CTL simulation. The detectability in the GHG and NAT simulations was less robust than that in the ALL simulations because the scaling factor CI was higher (error bars in Fig. 4). The GHG and NAT simulations could not determine that anthropogenic forcing has an effect on glacier mass loss (that is, the hypothesis of a zero scaling factor) at the 5% significance level. This may have been due to the small number of GCM ensembles used in those experiments. A sensitivity test revealed that the detection sensitivity of the ALL simulations was robust, even with few GCMs.

Our results do not reflect mass change in Asian mountain glaciers, because many were not included due to the limited availability of observations and the models’ inability to calculate reasonable mass changes in these regions. In addition, the results were skewed by large glaciers because we applied the optimal fingerprinting method to the area-weighted average of mass changes. Another potential limitation was the usage of reconstructed glacier mass instead of direct observations. Finally, uncertainty in a global glacier model that is used to calculate glacier mass forced by GCM simulations may be an additional confounding factor. Despite the aforementioned limitations, mass decreases were detectable during this period at larger spatial scales and the changes were robust, even compared to natural internal climate variability.

## Conclusions

An optimal fingerprinting technique was applied to observed glacier mass changes at five glaciers in Western Europe or to reconstructed mass changes of 85 widely distributed Northern Hemisphere glaciers. Recent increases in mass loss were seen in both observations and the ensemble means of ALL simulations at all of the five stations, leading to positive regression coefficients. On the other hand, the contributions of natural and anthropogenic forcing to the change varied among the five glaciers.

An area-weighted average of the reconstructed glacier masses over 85 glaciers also showed strong negative changes after the 1980s. This negative trend was obtained in the ALL and GHG experiments, but not in the NAT experiment, implying that the trend cannot be explained without considering the effects of anthropogenic radiative forcing on climate, as demonstrated by the mass change time series and regression coefficients (positive in ALL and GHG but negative in NAT when the uncertainty range was ignored). Therefore, our analysis of GHG experiments supports the findings of a previous study based on ALL and NAT simulations^9^. More importantly, we showed, for the first time, uncertainty ranges of the detection results by including a modeled glacier mass forced by a long-term GCM experiment. The results indicate that the effects of anthropogenic and natural influences cannot be fully attributed.

## Methods

### Model data processing and HYOGA2 simulation

We calculated annual changes in the mass of glaciers using a global glacier model, HYOGA2, which reasonably represents temporal variations of observed mass changes when observation-based atmospheric forcing is applied (Supplementary Fig. S2). Precipitation and temperature inputs to drive HYPGA2 were obtained from the global observation-based gridded climate dataset, H08^17^, or outputs of CMIP5 GCMs (Supplementary Table S2). These GCMs were selected according to the availability of daily precipitation and temperature data when we archived the data from the Program for Climate Model Diagnostics and Intercomparison (http://cmip-pcmdi.llnl.gov/cmip5/).

We ran HYOGA2 forced by daily GCM precipitation and temperatures and then calculated the multi-model ensemble mean masses, assuming that the GCM weights were equal. GCM precipitation and temperatures were first interpolated from the original horizontal resolution (specified in Supplementary Table S2) to the same global grid cell (30′ × 30′) and then the model bias was simply corrected using the climatology (1948–1980) of the monthly means of the global gridded climate dataset (H08)^17^ to preserve the historical trend. Bias in daily temperature by the GCMs was corrected by subtracting the difference in climatological monthly means for 1948–1980 between H08 and the GCMs, whereas bias in daily precipitation was corrected by employing the ratio of precipitation intensities between H08 and the GCMs at eight different intensity classes (1%, 5%, 10%, 15%, 20%, 25%, 30%, and >30% largest daily intensities) for 1948–1980. The wet day percentage at each grid of H08 for 1948–1980 was scaled by the ratio of each GCM’s wet day percentage for 1948–1980.

### Retrospective glacier simulation over 85 glaciers

For the global analysis, we selected 85 glaciers where the absolute bias in modeled mass change was less than 30% (Supplementary Information; Table S4). Then we analyzed the area-weighted mass balance of the 85 modeled and reconstructed glaciers. The 85 glaciers covered a large area in the Northern Hemisphere (Supplementary Fig. S1). The mass changes of 85 mountain glaciers were calculated using a retrospective HYOGA2 simulation for 1949–2003 forced by observation-based gridded meteorological data, H08^17^. We used this reconstruction as a pseudo-observation in the optimal fingerprinting method.

### Detection and attribution

The optimal fingerprinting method^10^,^11^ of detection and attribution was applied to the modeled and observed (or reconstructed) glacier mass changes. Observations of glacier mass balance (**y**) were expressed as a sum of scaled model-simulated fingerprint patterns (**X**) plus a climate noise term (**ε**) as


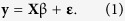


where β is a best estimate of the unknown scaling factor obtained using the total least squares method, and noise **ε** is assumed to be partly explained by natural internal climate variability calculated in the unforced control GCM simulations (CTL). **y** is either the observed glacier mass changes at 5 glaciers or the area-weighted mean of reconstructed glacier mass changes of 85 glaciers obtained from a retrospective HYOGA2 simulation (11 dimensional time vector, for a 5-year average of 55 years between 1949 and 2003). **X** is the multi-model mean of glacier mass change of the 5 or the area-weighted mean of 85 glaciers (11 dimensional time vector) obtained from HYOGA2 simulations that were forced by bias-corrected climate outputs of three GCM experiments (ALL, GHG and NAT). The 160 non-overlapping, 55-year chunks of CTL (Supplementary Table S3) were divided into two sets: one set of 80 chunks used to estimate the regression coefficient β and another set of 80 chunks used for the residual consistency test^10^ and the derivation of one-dimensional confidence intervals^6^.

Detection analyses were conducted in a full space of the time dimension (11, for a 5-year average of 55 years) after filtering through an empirical orthogonal function (EOF) analysis. Filtering was performed to improve the signal-to-noise ratio by normalizing observed and modeled data using natural internal climate variability, which provides a robust estimate of the inverse noise covariance^8^,^10^. We calculated the sum of the leading k eigenvectors, for 0 ≤ k ≤ 11, to confine the detection space to the 11 highest-ranked EOFs of the CTL. According to a previous study^6^, the optimal OLS^10^ approach was used to obtain 95% CIs.

In the optimal fingerprinting technique, the noise term in Equation (1) (**ε**) was assumed to be partially explained by the natural internal climate variability calculated in the unforced control GCM simulations (CTL). The noise derived from the CTL simulations denotes natural internal climate variability, which occurs without external forcing (e.g., the El Niño Southern Oscillation and Madden-Julian Oscillation). The variance covariance matrix of **ε** is given by the matrix **C**_N_:


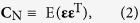


where E is the expectation operator and **C**_N_ is obtained from a set of CTL simulations. The total length of the CTL simulation (8,800 years) was comparable to those used in previous optimal fingerprinting studies. For example, ref. 3 used 4,300-year CTL data to analyze a 49-year observation, ref. 4 used 1,700-year CTL data to analyze a 91-year observation, and ref. 5 used 1,700-year CTL data to analyze a 75-year observation.

Assuming that **ε** is multivariate normal, the best (lowest variance) linear unbiased estimator (BLUE) of β can be obtained when **C**_N_ satisfies the Gauss-Markov assumption:


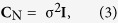


where σ^2^ is the variance of **ε** and **I** is the identity matrix. In an optimal fingerprinting method, the pre-whitening coordinate transformation **P** is introduced to **ε** as


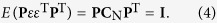


where **P** is the diagonal matrix of **C**_N_. The BLUE of β, 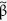, is estimated by differentiating (**Py**-**PX**β)(**Py**-**PX**β)^T^, which is the transformation of the right side of Equation (4) by applying Eq. (1) and **P** as


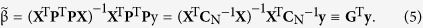


We followed the previous study^10^ to obtain one-dimensional 95% CIs using the OLS method. For the residual consistency test, we used two sets of 80 chunks of CTL simulations. The first was used to estimate the scaling factor β, and the second was used for the CI analysis of the estimated scaling factors. The one-dimensional confidence interval (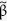 − β) was assumed to follow the Fisher distribution and was obtained from


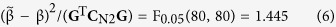


where **C**_N2_ is a matrix of the second set of CTL simulations and **G** and **G**^T^ are given by Equation (5).

### Residual consistency test

A residual consistency test was performed to evaluate the robustness of the derived natural internal climate variability from the CTL simulations. An F-test was applied to the variance of the two scaling factors (β_1_ and β_2_) obtained from the two different sets of chunks. An inverse linear interpolation method was applied to obtain the Fisher distribution for the 80 samples (1.445).

The variance of 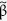, V(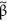), is given by


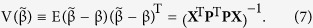


The test statistic F was calculated by dividing the variances of the two sets of β (β_1_ and β_2_) from the two samples:


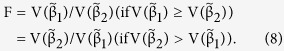


In total, 10 of 15 simulations of the three experiments (ALL, NAT, and GHG) over the 5 monitored glaciers passed the test (F < 1.445) (experiments in Fig. 1 without asterisks) and all three experiments of the 85 reconstructed glaciers passed the test (Fig. 4), implying that the 80 chunks of GCM control simulations were long enough to give consistent results.

## Additional Information

**How to cite this article**: Hirabayashi, Y. *et al*. Contributions of natural and anthropogenic radiative forcing to mass loss of Northern Hemisphere mountain glaciers and quantifying their uncertainties. *Sci. Rep.* **6**, 29723; doi: 10.1038/srep29723 (2016).

## Supplementary Material

Supplementary Information
