# Supplementary material for: Contributions of natural and anthropogenic radiative forcing to mass loss of Northern Hemisphere mountain glaciers and quantifying their uncertainties
Source: Sci Rep. 2016 Jul 20;6:29723. doi: 10.1038/srep29723 (PMC4951733; doi:10.1038/srep29723)
Supplement: Supplementary Information [file srep29723-s1.pdf]

Supplementary Information

Contributions of natural and anthropogenic radiative forcing to mass loss of Northern Hemisphere mountain glaciers and quantifying their uncertainties

Yukiko Hirabayashi, Kazunari Nakano, Yong Zhang, Satoshi Watanabe,
Masahiro Tanoue and Shinjiro Kanae

Evaluating reconstructed historical changes in glacier mass

Reconstructions of historical changes in glacier mass were obtained from a retrospective simulation of the global glacier model HYOGA2[13] forced by a gridded global climate data H08[17] for 1949–2003. HYOGA2 is a simple temperature-index-based global glacier model that calculates mass changes of individual mountain glaciers and ice caps at a 50m vertical resolution. The HYOGA2 provided reasonable glacier mass changes over stations with available observations[12]. The model was used to calculate global future mass loss from mountain glaciers and its contribution to sea level rise under the warming climate[12]. The basic model structure and model application to calculate future projection at a global scale were included in a reference.

We selected a total of 85 glaciers for which the absolute error of modeled mean annual mass changes in the simulation was < 30% (Supplementary Table S4). A comparison of observed and the reconstructed mass changes of the five selected glaciers showed that HYOGA2 reasonably represented temporal variation of glacier mass change (Fig. S2).

Evaluating detection and attribution using the reconstructed glacier mass change

Due to the limited available long-term observations of glacier mass change, we applied an optimal fingerprinting method to reconstructed mass of 85 widely distributed glaciers. To validate the methodology, we compared the regression coefficients of the ordinal (formal) optimal fingerprinting when using observed glacier masses at five glaciers or reconstructed glacier masses. The direction of β (positive or negative) was

the same in all of the 15 experiments (ALL, GHG and NAT experiments at five glaciers) except for one (GHG at Storbreen), indicating that reconstructed glacier mass can be a good alternative, when long-term observations are unavailable (Fig. 2 and Fig. S3).

References

18. Dyurgerov, M.B. & Meier, M.F. Glaciers and the changing earth system: A 2004 snapshot. Occasional Paper 58, Institute of Arctic and Alpine Research, University of Colorado at Boulder, Boulder, Colorado, USA. (2005).

Supplementary Tables

Table S1: Information on five monitored glaciers. The glacier information were taken from a reference[18].

No.	Name	Latitude	Longitude	Maximum altitude (m)	Minimum altitude (m)	Area (km^2)
1	Sarennes	45.07	6.10	3190	2830	0.5
2	Aletsch	46.30	8.02	4160	1556	127
3	Hintereisferner	46.48	10.46	3710	2426	8.0
4	Storbreen	61.34	8.08	1970	1380	5.3
5	Storgleciaren	67.54	18.34	1828	1125	3.2

Table S2: Summary of the GCMs selected for this study. The institution and model names were taken from http://cmip-pcmdi.llnl.gov/cmip5/availability.html. Size information was extracted from data headers.

GCMs from CMIP5	Institution	No. of grid
CCCma-CanESM2	Canadian Centre for Climate Modelling and Analysis, Canada	128 × 64
CMCC-CMS	Centro Euro-Mediterraneo per I Cambiamenti Climatici, Italy	192 × 96
CNRM-CM5	Centre National de Recherches Meteorologiques/Centre Europeen de Recherche et Formation Avancees en Calcul Scientifique, France	256 × 128
CSIRO-Mk3-6-0	Commonwealth Scientific and Industrial Research Organisation in collaboration with the Queensland Climate Change Centre of Excellence, Australia	192 × 96
GFDL-ESM2G	Geophysical Fluid Dynamics Laboratory, USA	144 × 90
HadGEM2-ES	Met Office Hadley Centre, UK	192 × 145
INM-CM4	Institute for Numerical Mathematics, Russia	180 × 120
IPSL-CM5A-LR	Institut Pierre-Simon Laplace, France	96 × 96
MIROC-ESM	Atmosphere and Ocean Research Institute, National Institute for Environmental Studies, and Japan Agency for Marine-Earth Science and Technology, Japan	128 × 64
MIROC5		256 × 128
MPI-ESM-LR	Atmosphere and Ocean Research Institute, National Institute for Environmental Studies, and Japan Agency for Marine-Earth Science and Technology, Japan<br>Max Planck Institute for Meteorology (MPI-M), Germany	192 × 96
MRI-CGCM3	Meteorological Research Institute, Japan	320 × 160
NCAR-CESM1-BGC	National Center for Atmospheric Research and Central Research Institute of Electric Power Industry, USA	288 × 192
NCC-NorESM1-M	Norwegian Climate Centre, Norway	144 × 96

Table S3: List of number of chunks for the preindustrial control (CTL) simulations and the availability of retrospective simulations with the anthropogenic forcing only (GHG) and the natural forcing only (NAT) scenarios of CMIP5 GCMs.

GCMs from CMIP3	# of 55-year chunks of CTL	GHG scenario	NAT scenario
CCCma-CanESM2	14	Y	Y
CMCC-CMS	9	N	N
CNRM-CM5	15	Y	Y
CSIRO-Mk3-6-0	9	N	N
GFDL-ESM2G	9	N	N
HadGEM2-ES	10	Y	Y
INM-CM4	9	N	N
IPSL-CM5A-LR	18	Y	Y
MIROC-ESM	11	Y	Y
MIROC5	12	N	N
MPI-ESM-LR	18	N	N
MRI-CGCM3	9	Y	Y
NCAR-CESM1-BGC	9	N	N
NCC-NorESM1-M	8	Y	Y
TOTAL	14 GCMs, 160 chunks	7 GCMs	7 GCMs

Table S4: List of 85 glaciers used to calculate reconstructed historical mass changes. The glacier information were taken from a reference[18]. The last column is the absolute mean error of annual mass balance between the reconstructed and observed mass change over the available observation period.

No.	Latitute	Longitude	Maximum altitude (m)	Minimum altitude(m)	area (km^2)	error (%)
1	45.53	6.56	3580	1480	33.0	0.12
2	45	6	3000	2782	0.2	0.01
3	45.11	6.1	3463	2650	3.0	-1.21
4	45.07	6.1	3190	2830	0.5	-0.27
5	46.37	8.24	3620	2125	17.4	-0.36
6	46.3	8.02	4160	1556	126.7	1.43
7	46.25	8.26	3272	2540	2.4	0.37
8	47.08	12.36	3050	2500	1.5	-0.55
9	47.02	13	3120	2380	1.1	-0.45
10	46.51	10.06	3160	2290	2.6	-0.94
11	45.31	7.26	3170	2900	0.8	-0.44
12	46.97	11.23	3125	2620	1.1	-0.39
13	61.48	7.21	1630	1160	11.9	-4.53
14	66.43	14.27	1300	900	1.8	-1.05
15	66.41	14	1580	520	59.16	-2.16
16	66.27	13.39	1160	588	2.6	-1.09
17	70.1	21.45	1050	280	3.7	-1.8
18	61.45	5.39	1380	890	4.4	0.41
19	61.45	5.41	1320	925	2.9	-1.28
20	61.00	6	1570	1460	1.5	-0.71
21	61.33	8.26	2280	1630	8.71	-3.75
22	61.32	8.3	2200	1570	9.0	-0.32
23	61.34	8.08	1970	1380	5.3	-2.22
24	61.39	8.36	2300	1850	2.4	-1.05
25	61.5	7.16	1730	1130	4.2	-1.9
26	60.09	6.29	1650	1100	8.9	-0.01
27	60.1	6.43	1610	1065	4.5	-1.46
28	68.05	18.41	1740	1340	4.0	-2.05
29	67.54	18.34	1828	1125	3.2	-1.34
30	67.54	18.33	1700	1071	3.9	-1.7
31	64.43	-19.03	1750	750	51.3	-18.9
32	64.55	-18.5	1800	860	90.6	-3.38

33	64.48	-18.35	1800	620	248.8	-3.39
34	64.42	-14.53	1240	820	1.8	1.08
35	64.25	-17.2	1760	1380	160.0	28.9
36	42.39	0.38	3180	2790	0.5	-0.03
37	43.12	42.46	3798	2700	3.1	0.19
38	43.12	42.46	3600	3300	0.4	-0.22
39	43	42	4342	3100	3.3	0.41
40	42.55	43.4	4460	2200	9.7	0.03
41	43.1	41.4	3240	2270	2.7	-0.98
42	43.18	42.28	4300	2950	3.8	-1.49
43	50.05	87.44	4043	2559	6.0	-0.07
44	50.06	87.42	3550	3025	0.75	-0.02
45	50	87	3867	2480	16.81	-0.3
46	45.05	80.14	4464	3126	2.8	-0.04
47	30.33	79.54	5150	4240	2.6	-2.04
48	35.4	94.11	5520	4805	1.1	-0.46
49	33.1	92.08	5926	5380	1.8	-0.01
50	39.14	97.54	5145	4310	3.0	0.07
51	37.33	101.45	5024	4200	1.4	-0.07
52	39.14	98.34	5262	4420	1.6	-0.03
53	43	77.06	4219	3414	2.7	-0.98
54	43	77	4220	3450	1.7	-0.65
55	43	77	4150	3450	1.4	-0.57
56	42	79	5520	3400	84.1	-0.97
57	42.06	78.18	4829	3293	4.6	-1.8
58	41.5	78.12	4980	3780	11.4	-0.48
59	41.47	77.47	4496	3895	1.3	-0.59
60	78.05	17	1200	30	199.5	8.39
61	78	14.05	600	5	17.8	0.29
62	77.05	15.24	810	27	28.0	-11.71
63	69.17	-143.5	2700	1350	7.4	0.05
64	63.26	-146.47	3500	880	46.0	-0.54
65	63.31	-147.23	3591	845	311.0	0.82
66	63.14	-145.28	2460	1165	19.3	-0.71
67	63.16	-145.3	2100	1325	2.23	-0.93
68	63.31	-146.57	3600	815	323.0	0.63
69	60.22	-148.54	1700	400	17.62	-6.51

70	60.55	-145.13	1590	110	54.1	-1.04
71	60	-139.18	2492	53	28.0	-10.4
72	59.41	-137.46	1980	670	27.9	-0.07
73	58.4	-134.12	2160	20	671.0	21.63
74	56.56	-130.59	2190	610	91.9	0.9
75	51.26	-124.55	2740	1480	10.51	-4.53
76	50.26	-122.36	2610	1860	3.5	-1.57
77	50.52	-123.35	2750	1520	25.35	1.2
78	50.48	-123.25	2500	2010	6.5	-0.06
79	51.4	-116.32	3185	2125	11.8	-4.64
80	47.49	-123.41	2320	1280	4.2	0.25
81	46.48	-121.44	2430	1400	4.6	-2.66
82	47.58	-121.21	2080	1840	0.9	0.19
83	48.39	-121.11	2390	1780	0.7	0.05
84	48.34	-121.07	2399	1729	1.5	0.04
85	48.59	-121.15	2698	2088	1.1	0.64

Supplementary Figures

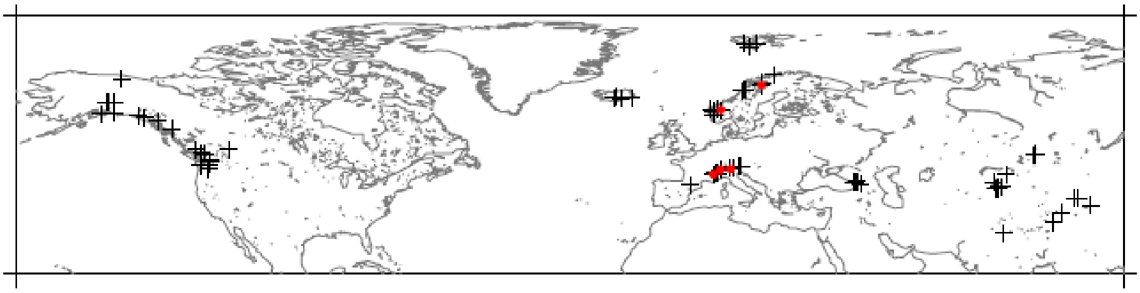

Figure S1: Locations of 5 monitored glaciers (red dot) and 85 selected glaciers (black cross). This figure was created using Python 2.7.2.

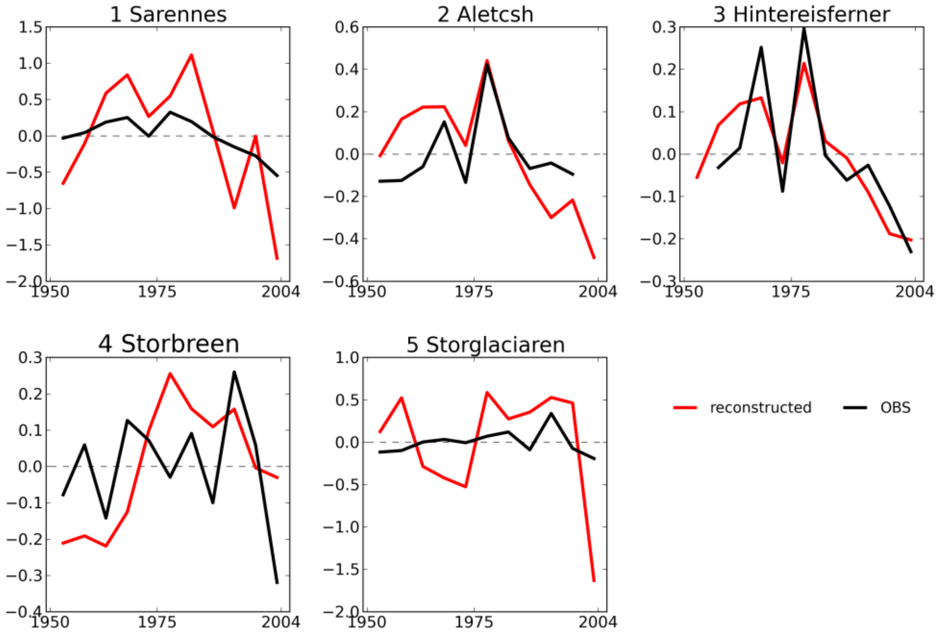

Figure S2: Comparison of observed and reconstructed mass balance anomalies of 5 monitored glaciers. This figure was created using Python 2.7.2.

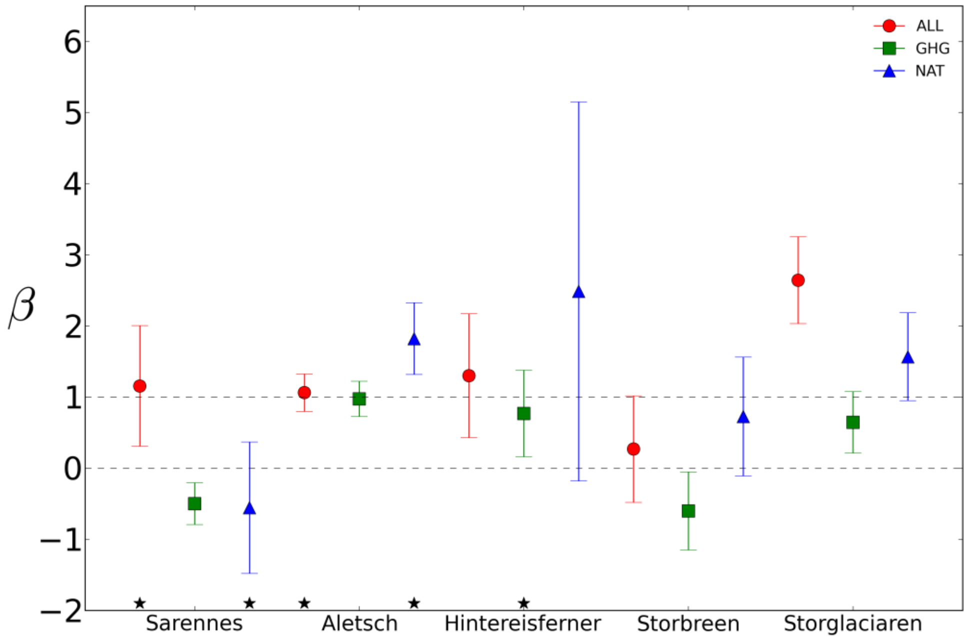

Figure S3: Same as Figure 2 but for reconstructed glacier mass as a pseudo-observation. This figure was created using Python 2.7.2.

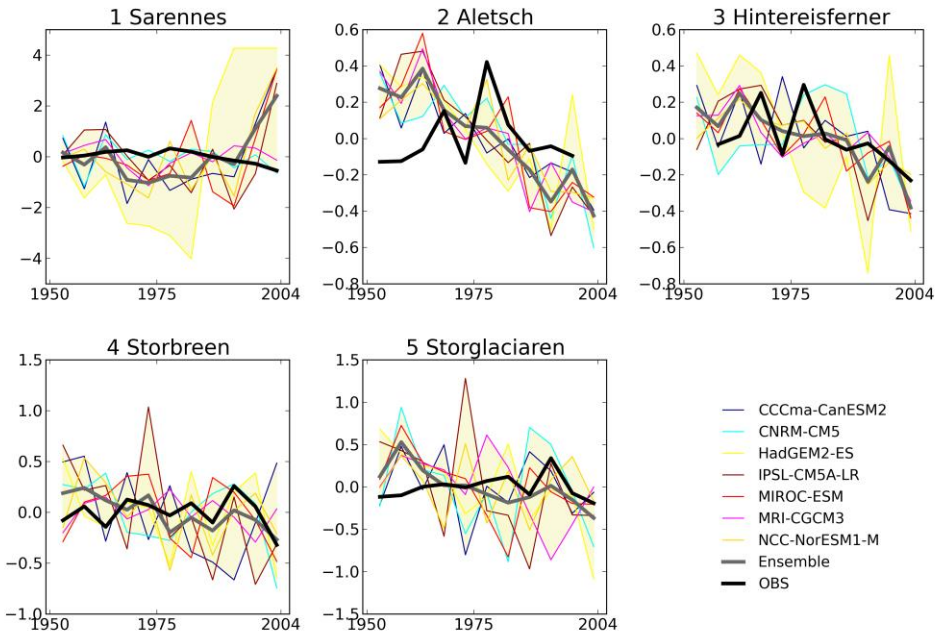

Figure S4: Same as Figure 1, but for the GHG scenario. This figure was created using Python 2.7.2.

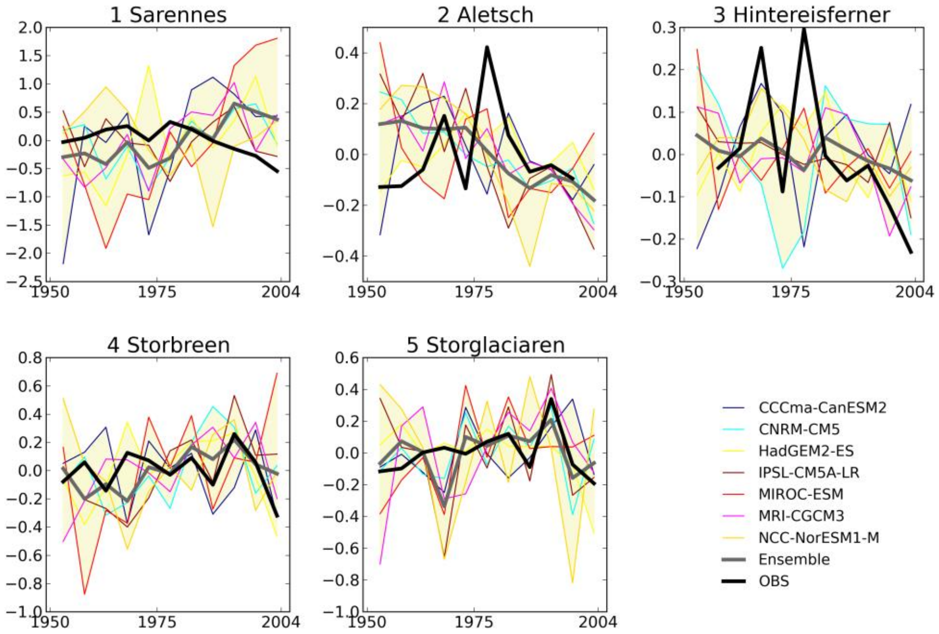

Figure S5: Same as Figure 1, but for the NAT scenario. This figure was created using Python 2.7.2.
